# Comparison of COVID-19 Vaccine Approvals at the US Food and Drug Administration, European Medicines Agency, and Health Canada

**DOI:** 10.1001/jamanetworkopen.2021.14531

**Published:** 2021-06-25

**Authors:** Mark P. Lythgoe, Paul Middleton

**Affiliations:** 1 Department of Surgery and Cancer, Imperial College London, Hammersmith Hospital, London, United Kingdom; 2 Department of Metabolism, Digestion and Reproduction, Imperial College London, St Mary’s Hospital, United Kingdom

## Abstract

This qualitative improvement study investigates COVID-19 vaccine approvals at 3 medicine regulatory agencies in the US, EU, and Canada, characterizing and contrasting regulatory review times, and analyzing the clinical evidence supporting authorization.

## Introduction

On the 1-year anniversary of the World Health Organization (WHO) declaring COVID-19 a global pandemic, the development and rollout of safe and effective vaccines has fueled optimism for greater pandemic control. During the COVID-19 pandemic, medicine regulators have faced significant pressure from both the public and governments to expedite vaccine approval, while grappling with the challenges of novel vaccine clinical development and ensuring public trust and confidence in COVID-19 vaccines.^1^ Recognizing the gravity of this public health emergency, medicine regulators have introduced or activated accelerated mechanisms for restricted approval or permitted use of unapproved medical products in predefined circumstances (eg, Emergency Use Authorizations [EUA]).^2^ We investigated COVID-19 vaccine approvals at 3 medicine regulatory agencies, the US Food and Drug Administration (FDA), European Medicines Agency (EMA), and Health Canada (HC), characterized and contrasted regulatory review times, and analyzed the clinical evidence supporting authorization.

## Methods

This quality improvement study did not require institutional review board approval or informed consent because the collected data are based on publicly available information, and no individual patient information was analyzed. This study followed the Standards for Quality Improvement Reporting Excellence (SQUIRE) reporting guideline.

We identified all COVID-19 vaccines approved for use by searching regulatory websites for the FDA, EMA, and HC on March 11, 2021. Using publicly available documents, we identified the regulatory submission date, the approval or authorization date, and the nature of authorization for each vaccine. All clinical trials considered or named in regulatory approval reports were identified by the ClinicalTrials.gov identifier, and variables, including use of masking, randomization, comparator groups, and primary end points, were recorded. Furthermore, we identified pivotal efficacy trials either identified by regulators or using methods outlined previously.^3^ When variability occurred between medicine regulators in the number of clinical trials supporting vaccine authorization, the higher number was included. Finally, we identified the patient numbers in prelicense regulatory safety assessments for each vaccine, including both those receiving a vaccine and those receiving a placebo/active comparator. Data analyses were performed using Microsoft Excel Version 16.47, (Microsoft Corporation).

## Results

We identified 4 different COVID-19 vaccine authorizations overall: 3 by the FDA and 4 by both the EMA and HC. The equivalent ChAdOx1 nCoV-19 recombinant vaccine, COVISHIELD, was not included. The median review time from initial submission to approval was similar for the FDA (21 days; range, 18-23) and EMA (22 days; range, 18-37), but longer for HC (84 days; range, 61-148). However, submissions to HC were sent a mean (SD) of 65 (26) days earlier than the corresponding earliest submission to either the FDA or EMA, negating any meaningful delay in vaccine authorization (Table 1).

**Table 1.** COVID-19 Vaccine Approval by Regulatory Agency

COVID-19 Vaccine	US Food and Drug Administration			European Medicines Agency			Health Canada		
	Submission date	Authorization date^a^	Time for review, (d)	Submission date	Authorization date^b^	Time for review, (d)	Submission date	Authorization date^c^	Time for review, (d)
Pfizer-BioNTech (BNT162b2)	11/20/2020	12/11/2020	21	11/30/2020	12/21/2020	21	10/09/2020	12/09/2020	61
Moderna (mRNA-1273)	11/30/2020	12/18/2020	18	11/30/2020	01/06/2021	37	10/12/2020	12/23/2020	72
Janssen (Ad26.COV2.S)	02/04/2021	02/27/2021	23	02/16/2021	03/11/2021	23	11/30/2020	03/05/2021	95
Oxford or AstraZeneca (ChAdOx1)	NA	NA	NA^d^	01/11/2021	01/29/2021	18	10/01/2020	02/26/2021	148
Median review time, (range), d			21 (18-23)			22 (18-37)			84 (61-148)

Abbreviation: NA, not applicable.

^a^ Vaccine approved under Emergency Use Authorization.

^b^ Vaccine approved under Conditional Marketing Authorization.

^c^ Vaccine authorized with specific terms and conditions.

^d^ Vaccine not approved.

Each COVID-19 vaccine analyzed was supported by a median of 4 (range, 2-5) clinical trials with 1 (range, 1-2) clinical trial considered pivotal to authorization. Among the 14 clinical trials contributing to vaccine authorization, 12 (85.7%) were randomized, 9 (64.2%) were double-masked, 8 (57.1%) had primary clinical end points, and 3 (21.4%) used an active comparator. For the 5 clinical trials that were deemed pivotal, 5 (100%) were randomized and had primary clinical end points, 3 (60%) were double-masked, and 2 (40%) used an active comparator. The presubmission phase 2 and 3 trial safety databases for all 4 vaccines included 135 464 patients, with a median patient number per vaccine authorization of 33 818 (range, 12 021-43 783) with 50% receiving a COVID-19 vaccine and the remainder placebo or active comparator (Table 2).

**Table 2.** Characteristics of COVID-19 Vaccine Clinical Trials Considered by Medicine Regulators

Feature	Median (range)
Clinical trials supporting vaccine approval, No.	4 (2-5)
Pivotal efficacy trials supporting vaccine approval, No.	1 (1-2)
Clinical trial, No. (%)	
With randomization	12 (85.7)
With double-masking	9 (64.2)
With active comparator^a^	3 (21.4)
With placebo comparator	9 (64.2)
With primary clinical end point	8 (57.1)
Patients in Phase 2/3 safety database, median (range), No.	
Total	33 818 (12 021-43 783)
Receiving COVID-19 vaccine	16 993 (12 021-21 895)
Receiving active/placebo comparator	16 826 (11 724-21 888)

^a^ All studies used the Meningococcal group A, C, W-135, and Y conjugate vaccine as an active comparator.

## Discussion

COVID-19 vaccines have been authorized for use by regulators at unprecedented speed. Between 2010 and 2020, the FDA approved 21 new vaccines for use, with a median review time from submission to approval of 12 months.^4^ By permitting EUA for COVID-19 vaccines, the median review time was 21 days during the COVID-19 pandemic. Regulators have adopted new pathways and frameworks that allow for rapid vaccine authorization in specific circumstances and have acknowledged the need for more data regarding safety and efficacy to permit full approval.^2^ Medicine regulators are engaging with vaccine developers earlier; for example, HC has developed fast-track approval processes specifically for COVID-19 vaccines, which begins the review process earlier and allows evidence to be reviewed as it becomes available.^5^ Furthermore, increasing global regulatory harmonization is enabling the robust and timely approval of COVID-19 vaccines.^6^ With the potential challenges that new variants of SARS-CoV-2 may bring, the COVID-19 vaccine armamentarium will likely need further additions and requires prompt regulatory approval to ensure that we can meet the continuing challenges brought by the global pandemic. This study has limitations, including data availability in study documents, differences in authorization types, and variability in medicine regulator processes and terminology.
